# Implementing risk stratification to the treatment of adolescent substance use among youth involved in the juvenile justice system: protocol of a hybrid type I trial

**DOI:** 10.1186/s13722-019-0161-5

**Published:** 2019-09-06

**Authors:** Matthew C. Aalsma, Allyson L. Dir, Tamika C. B. Zapolski, Leslie A. Hulvershorn, Patrick O. Monahan, Lisa Saldana, Zachary W. Adams

**Affiliations:** 1 Department of Pediatrics, Section of Adolescent Medicine, Indiana University School of Medicine, 410 West 10th Street Suite 2000, Indianapolis, IN 46202 USA; 2 Adolescent Behavioral Health Research Program, Indiana University School of Medicine, Indianapolis, IN USA; 3 Department of Psychology, Indiana University Purdue University Indianapolis, Indianapolis, IN USA; 4 Department of Psychiatry, Indiana University School of Medicine, Indianapolis, IN USA; 5 Department of Biostatistics, Indiana University School of Medicine, Indianapolis, IN USA; 6 Oregon Social Learning Center, Eugene, OR USA

**Keywords:** EPIS, Implementation, Adolescent substance use, Brief intervention, Justice-involved youth, Screening, Hybrid design, Effectiveness

## Abstract

**Background:**

Youth involved in the juvenile justice system (YIJJ) have high rates of substance use problems; however, rates of YIJJ engagement in substance use services is low. Barriers to service engagement include lack of appropriate screening and connection to services by the juvenile justice system, as well as lack of resources for delivering evidence-based treatment in community-based settings. To address these barriers, this paper describes a protocol for a type 1 hybrid design to (1) implement universal substance use screening for YIJJ; (2) implement and evaluate the feasibility and effectiveness of a brief, three-session substance use interventions based in motivational interviewing for youth with mild/moderate substance use: Teen Intervene (an individual-based intervention); (3) implement ENCOMPASS, an evidence-based substance use intervention based in motivational enhancement and cognitive behavioral therapy for youth with severe substance use; and (4) evaluate facilitators and barriers to implementing these interventions for mild to severe substance use among YIJJ in community mental health centers (CMHC).

**Methods/design:**

Using a hybrid type 1 clinical effectiveness-implementation design, we will collaborate with CMHCs and juvenile justice in two rural Indiana counties. Guided by the EPIS (exploration, preparation, implementation, sustainability) framework, we will measure factors that affect implementation of substance use screening in juvenile justice and implementation of substance use interventions in CMHCs utilizing self-reports and qualitative interviews with juvenile justice and CMHC staff pre- and post-implementation. YIJJ with mild/moderate substance use will receive a brief interventions and YIJJ with severe substance use will receive ENCOMPASS. We will measure the effectiveness of a brief and comprehensive intervention by assessing changes in substance use across treatment. We anticipate recruiting 160 YIJJ and their caregivers into the study. We will assess intervention outcomes utilizing baseline, 3-, and 6-month assessments.

**Discussion:**

Findings have the potential to improve screening and intervention services for YIJJ.

## Background

Youth involved in the juvenile justice system (YIJJ) present with substance use disorders (SUDs) and mental health problems at significantly higher rates than non-offending peers in the community [1–4]. Though estimates vary by population characteristics and assessment criteria [5], up to 80% of justice-involved youth report lifetime substance use; moreover, between up to 40% of justice-involved youth meet criteria for a SUD [3, 6, 7], and a significant portion of youth offenders suffer multiple comorbid mental health disorders and SUDs [2, 8]. Arrested youth also initiate substance use earlier than other adolescents, which increases risk of developing more severe substance use problems [5]. While cannabis and alcohol are the most widely used substances among both justice-involved and non-justice involved adolescents, justice-involved youth use more dangerous drugs, such as opioids, at higher rates compared to their non-justice-involved peers [9]. Despite this significant behavioral health burden, YIJJ access behavioral health (mental health and substance use) services at low rates, and at much lower rates than non-offending peers [10]. For example, in one large scale study of youth offenders, well under 20% of detained youth were engaged in behavioral health treatment at 60 days following release to the community [6]. Effective evidence-based interventions targeting behavioral health among YIJJ exist [11–17]; however, only a relatively small number of communities have access to these programs, contributing to the findings that of the small sample of YIJJ who engage in behavioral health treatment, most do not receive evidence-based treatment [14]. The goal of this manuscript is to describe a protocol to implement substance use screening in juvenile justice settings and evidence-based substance use interventions in community mental health centers targeting YIJJ.

### Barriers to engagement in substance use and behavioral health treatment

There are multiple barriers that prevent YIJJ from engaging in substance use—and other behavioral health—treatment; however, for the purposes of the proposed study, we highlight barriers at the level of the juvenile justice system related to screening and identification of YIJJ in need of services, and barriers at the community level in regards to providing appropriate and evidence-based services.

#### Substance use screening in the juvenile justice system

First, there is a lack of systematic mental health and substance use screening in the juvenile justice system [18]. Given the high rates of substance use and mental health problems among YIJJ, as well as the higher risk of recidivism among YIJJ with substance use and mental health problems, screening and identifying those in need of services is an important task for juvenile justice staff [6, 18–20]. Although juvenile probation officers (JPO) typically function as gatekeepers who help connect YIJJ with appropriate services, there is often a significant lag time between YIJJ’s release from detention and their first contact with a JPO, which leads to delays in service connection and engagement. Moreover, arrest and detention can be viewed as a “crisis event” for youth during which time their motivation for seeking behavioral health services is high; therefore, screening and identification of service needs and connection to services is crucial during the initial intake period following arrest [21]. Thus, we propose to implement universal substance use screening in juvenile justice settings during initial intake following arrest in order to increase identification of YIJJ in need of services and in turn increase referral and connection to appropriate services in community-based settings.

### Evidence-based substance use interventions in community mental health centers

Although evidence-based interventions (EBI) for substance use targeting YIJJ exist [22], high-quality EBIs often are not available in community mental health centers (CMHC). In addition to lack of dissemination and implementation of EBIs in CMHCs, there is also a shortage of behavioral health providers and clinicians in community-based settings to provide these services, and caseloads often are too large to implement intensive treatments [23, 24]. Therefore, there is a need for implementation of cost-effective, feasible EBIs that appropriately address substance use with YIJJ in community-based settings.

In accordance with the substance use care continuum [25], we propose to implement both brief and more comprehensive substance use interventions in CMHCs in order to address the heterogeneity in substance use severity. Implementing EBIs for varying levels of substance use risk ensures that lower risk individuals are not assigned to a higher level of care than necessary, as this creates a burden on the individual receiving treatment and reduces treatment engagement. Further, brief interventions are also cost-effective, and since they can be implemented by professionals with a range of training background, this limits the burden on community-based behavioral health providers by distributing workload across all personnel [26].

We propose to examine the effectiveness of an individual-based brief intervention, Teen Intervene [27, 28], which is based in motivational interviewing and have been show to effectively reduce mild to moderate substance use among adolescents and, specifically, among YIJJ [29]. Teen Intervene is a manualized, three- to six-session individual-based treatment targeting youth substance use that utilizes principles of motivational interviewing, cognitive-behavioral therapy, and self-change principles [27, 28]. Teen Intervene has been shown to reduce substance use and increase motivation to change substance use among adolescents [28] and court-involved adolescents [29] with mild to moderate substance use.

In addition to implementing a brief intervention, we also will implement a more intensive, comprehensive EBI for YIJJ with more severe substance use problems. There are several empirically supported outpatient treatment options for adolescents with problematic substance use and more severe substance use disorders [30], including cognitive behavioral therapy (CBT), motivational enhancement treatment (MET), and contingency management (CM). Moreover, these EBIs have been shown to be effective in reducing substance use when delivered in combination, such as MET combined with CBT and contingency management (MET/CBT + CM). One such integrated intervention model, ENCOMPASS, is a manualized outpatient intervention that combines MET/CBT + CM and pharmacotherapy to treat adolescents with co-occurring substance use and common mental health disorders (e.g., depression, attention-deficit/hyperactivity disorder). ENCOMPASS has been implemented in three randomized controlled clinical trials in youth who met criteria for an average of three substance use disorders and two to three psychiatric disorders [31–33]. Collectively, results demonstrated significant reduction in both substance use and mental health disorder severity—with outcomes comparable or superior to outcomes from trials for separate or sequential substance use or mental health treatments. ENCOMPASS also entails pharmacotherapy, which may include medication assisted treatment for more severe substance use, such as opioid use or alcohol. YIJJ are more likely to use opioids and have more severe substance use problems compared to non-offending peers [9]; thus, implementation of EBIs that include medication assisted treatment is important given the effectiveness of medication assisted treatment for adolescents with opioid use disorder [34–36].

### Overview of study design and objectives

In order to address service connection and availability of community-based substance use EBIs for YIJJ, we propose to partner with community mental health centers and juvenile justice systems in two Midwestern counties to: (1) implement universal substance use screening for probation youth (at the point of arrest) to determine substance use risk level and appropriate treatment needed; (2) implement and evaluate the feasibility and effectiveness of a brief substance use intervention for youth with mild/moderate substance use problems in community mental health centers; and (3) implement a comprehensive adolescent-specific evidence-based substance use treatment in community mental health centers. We propose to utilize a hybrid type 1 clinical-effectiveness-implementation trial [37] in order to evaluate facilitators and barriers to implementing this approach as well as test the effectiveness of these interventions in reducing substance use among YIJJ.

## Method

### Implementation model

**Fig. 1:**
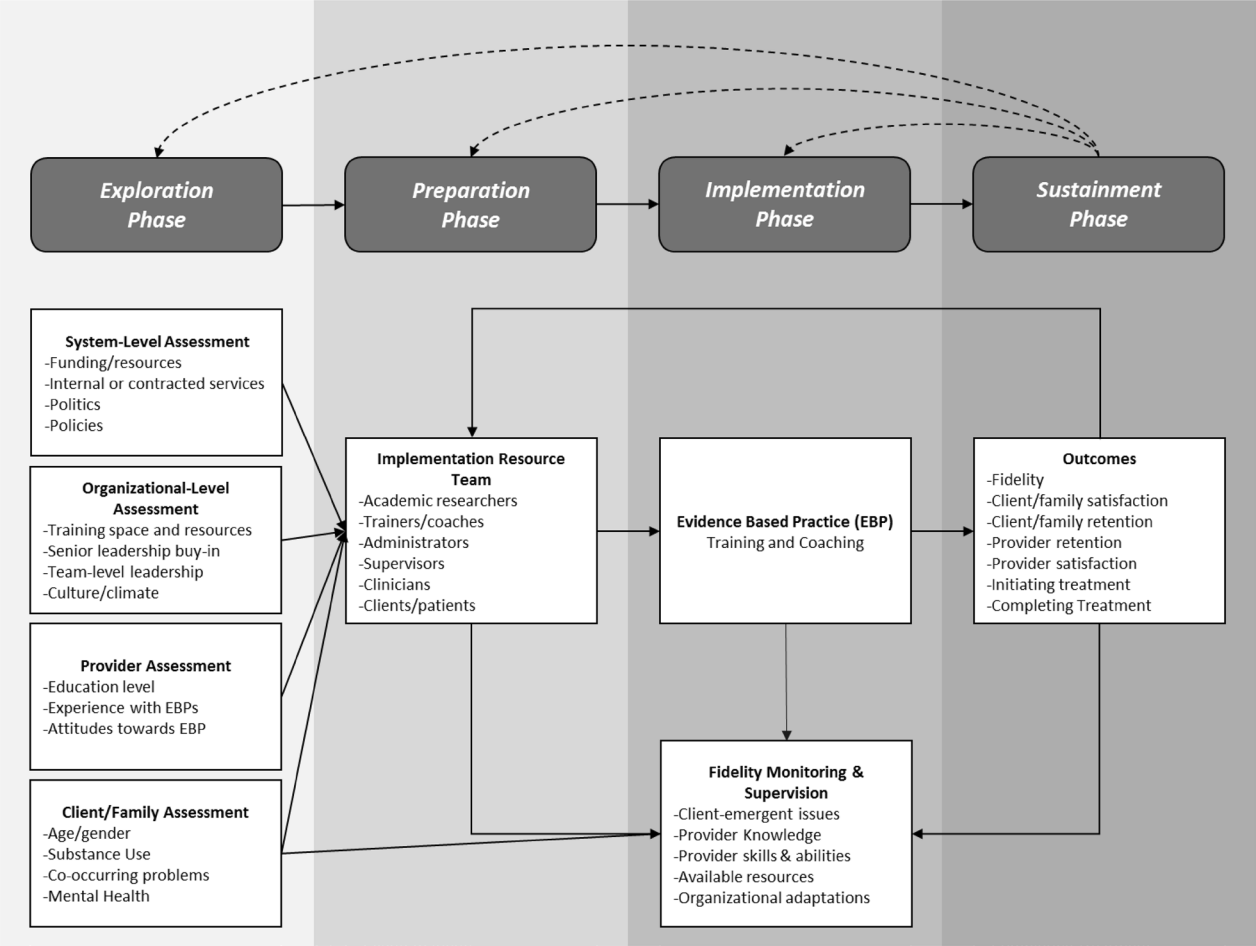
EPIS implementation model

Implementation will be guided by the Exploration, Preparation, Implementation, and Sustainment (EPIS) framework to study how best to implement the bundled treatment approach in juvenile justice settings and CMHCs [38]. We will utilize the Stages of Implementation Completion (SIC) implementation approach to measure inner and outer context factors that affect implementation of best-practices [38–41]. The outer context, in this case, includes state and local policies regarding funding and provision of addiction services for youth in general and, specifically, for justice involved youth. Inner context includes the organizations climate, culture, staffing as well as characteristics of juvenile justice and community mental health center (CMHC) staff towards adolescents (see Fig. 1 for illustration of EPIS model). The EPIS framework also explicitly identifies project phases, including exploratory phases, which is useful in the conceptualization of our pilot project. To guide and assess implementation we will collect data from self-report surveys and interviews conducted with substance use providers, parents, youth and court personnel across the study period.

### Study setting

Two counties in a Midwest state were chosen based on high rates of substance-related arrests and rates of substance use among adolescents in the community. Counties were chosen given their small size as well as the presence of one primary community mental health center (CMHC) that is designated to serve the community. This allows for feasibility of building and strengthening relationships between juvenile justice and community mental health professionals/agencies.

### Participants

#### YIJJ and caregivers

We will recruit YIJJ aged 14–17 in one of two counties who are either detained in the detention center and returning to the community or currently in the community. Eligible youth will be those who are identified as having a history of substance use, based on screening done by the juvenile justice staff (see below for explanation). Both youth and their legal guardian must agree to participate. Only youth and caregivers who are fluent in English will be eligible to participate.

#### Community partner participants

Community partner participants will be clinicians, case managers, and supervisors working in the CMHC in each county (range n = 10–30). Bachelor’s level case managers from each CMHC site (n = 2 case managers for each county) will be recruited to act as intervention specialists. They will be trained in the brief intervention and provide treatment to YIJJ and their caregivers. Master’s level licensed clinicians from each CMHC (n = 2 for each county) will be trained in the comprehensive substance use intervention and provide treatment to YIJJ.

The juvenile justice system in each county have agreed to participate and implement substance use screening. Designated juvenile probation officers/intake officers will be identified and will be responsible for conducting universal substance use screening with all youth coming through the detention center at intake.

In addition to these primary individuals who will provide interventions, we will seek participation from other personnel (e.g., juvenile justice intake staff, CMHC supervisors) who are involved in the implementation and sustainability of the interventions to complete qualitative interviews and quantitative measures to assess feasibility and implementation in these settings.

### Training and fidelity monitoring

#### Juvenile justice substance use screening

We will conduct a one-time training for juvenile probation officers at each of the JJ system sites to train them on the substance use screening tool (see below for details). Training is minimal compared to the intervention training given the ease/usability of the measure.

#### Interventions

Prior to the start of the trial, therapists and intervention specialists at each of the two sites will be trained by respective intervention training teams (i.e., training team for Teen Intervene and ENCOMPASS). Bachelor’s level brief intervention specialists will be trained in the brief interventions. For the purposes of wider dissemination of these evidence-based brief interventions, non-research providers at each CMHC also will have the opportunity to receive initial training. Following a one-time initial training, brief intervention specialists will have monthly meetings with the training team for ongoing training, consultation, and fidelity monitoring. Master’s level therapists who will be designated as ENCOMPASS therapists at each of the sites will be trained in ENCOMPASS by the ENCOMPASS master trainers. Following initial in-person training, ENCOMPASS therapists will engage in weekly meetings with the ENCOMPASS trainers for ongoing training, consultation, and fidelity monitoring. Psychotropic medication prescribers are also engaged in the weekly meetings and supervision is provided by an adolescent addiction psychiatrist.

##### Fidelity monitoring

Fidelity monitoring for the brief intervention and ENCOMPASS will be conducted by the respective intervention trainers.

*Teen Intervene* Brief intervention specialists will complete self-rating checklists following each session and also audiotape sessions to be rated by Teen Intervene trainers for fidelity [28]. Therapists and raters will complete the same checklists, which include items related to module-specific activities as well as items related to adherence to principles of motivational interviewing (e.g., reinforcing change talk) and cognitive behavioral techniques (e.g., assigning homework). Items are rated on a 5-point scale, with higher scores denoting greater fidelity/adherence and competency in skills. Mean scores of 3 across all items denote sufficient competency and fidelity.

*ENCOMPASS* ENCOMPASS therapists will complete self-rating treatment adherence fidelity checklists following each session as well as audio record sessions to be reviewed by ENCOMPASS trainers for fidelity checks. The same checklist will be used by therapists and raters to measure adherence and consists of six items related to principles of cognitive behavioral therapy six items related to principle motivational interviewing and motivational enhancement techniques, as well as items related to module-specific tasks and activities. Therapists self-rate their performance for each session in each of these areas on a 6-point scale (0 = not completed, 1 = low skill level to 5 = high skill level); thus, fidelity to the treatment can be quantified as an overall mean score for each session. Overall mean scores of 3 or above on the self-rating forms and/or trainer ratings of audiotaped sessions will be considered meeting minimum fidelity/adherence standards [32, 33].

### Procedures

#### Screening and referral

Juvenile probation officers will screen all youth who come through the intake process at the detention center using the CRAFFT [42]. The CRAFFT consists of six yes or no questions regarding substance use (e.g., do you ever use alcohol or drugs to relax; has family ever been concerned about your alcohol or drug use) as well as questions regarding frequency of past year substance use, with higher scores (range 0–6) denoting more severe substance use problems (see Additional file 1: Appendix S1 for complete measure). The CRAFFT has been shown to be an effective clinical tool in identifying substance use risk level among children and adolescents [41]. The research team will be notified of any youth scoring at least a 1 on the CRAFFT measure. Participants also will be notified of the opportunity to participate in an intervention and will be told that they might be contacted regarding voluntary participation in a research study. The research team will contact potentially eligible participants.

#### Enrollment and baseline assessment

Following initial recruitment, research assistants will complete consent/assent with the caregiver/youth at their home or preferred location. As part of the consent process, participants will be told that this is an intervention study and that they might be assigned to different interventions. Participants and their caregivers also will complete a baseline assessment, which includes self-report measures of demographics, family functioning, mental health, substance use (see Table 1 for overview of all measures).

**Table 1:** Overview of data collection

	Informant	Content	Time of Completion
*Implementation measures*			
System-level implementation	CMHC staff, JJ staff, supervisors	Implementation Citizenship Behavior Scale [47], Implementation Climate Scale [48], Implementation Leadership Scale [49], Organizational Readiness to Implement Change (ORIC) [50] Qualitative interviews	Pre-, post-implementation
	JJ staff, supervisors	Qualitative interviews	Pre-, post-implementation
Intervention fidelity monitoring/adherence	Intervention providers	Brief intervention fidelity self-rating checklists ENCOMPASS treatment adherence checklist	Weekly
	Intervention trainers	Fidelity monitoring checklists based on therapist recorded sessions	Monthly
Treatment satisfaction	Parent, youth	Service Satisfaction Scale [51]	1-month post-intervention
Treatment response	Youth	BSCQ [42], FCU Clinical Monitoring Report [43], BSTAD [52]	Post 1st and 2nd intervention
*Participant*-*level measures*			
Demographics	Youth, parent	Family affluence [53], Family characteristics	Baseline
Substance use	Youth	CRAFFT [50], BSCQ [51], BSTAD, AUDIT-C [54]	Baseline, 3-, 6-months
Family functioning	Youth, parent	Parental Monitoring Scale [55]	Baseline, 3-, 6- months
Health service use history	Parent		Baseline, 3-, 6-months
Psychosocial functioning	Youth, parent	Difficulties in Emotion Regulation Scale [56], Peer Conflict Scale [57], Hope & Life Satisfaction Scales [58]	Baseline, 3-, 6-months
Mental health	Youth	K-CAT (computerized child diagnostic evaluation; [59])	Baseline, 6-months

All measures are self-report unless noted otherwise

#### Risk level determination

Based on the youth’s initial CRAFFT score as well as their scores on initial baseline measures collected after consent at the first/baseline visit, youth will be categorized as either low to mid-risk or high-risk based on criteria from the American Society of Addiction Medicine (ASAM). ASAM criteria utilizes six dimensions of an individual’s substance use and overall functioning to determine appropriate level of care [25]. We will determine risk level based on youth’s pattern of substance use, such as frequency and quantity of use, as well as problematic use based on DSM-5 substance use disorder symptoms. Table 2 describes specific risk determination criteria we will determine based on assessment. Generally, high-risk youth will be those who have identified past year repeated use of more lethal substances with a high risk for overdose and withdrawal potential (e.g., opioids, methamphetamine) and those who endorse three or more DSM-5 SUD symptoms, which is consistent with a moderate to severe SUD. All other youth initially will be randomized to one of the brief interventions. Based on preliminary data collected from each county, it is estimated that about 10% of youth will be in the high-risk group.

**Table 2:** Risk level determination

	Brief intervention (low/mid risk)	Comprehensive intervention (high risk)
Substance use frequency	Less than weekly use of cannabis, alcohol, nicotine	Weekly or more frequent use of any substance Past year repeated use of lethal substance (opioids, methamphetamine, benzodiazepines)
Problematic substance use	Minimal problems or consequences of use ≤ 2 DSM-5 SUD symptoms	History of overdose Report of experiencing withdrawal ≥ 3 DSM-5 SUD symptoms

Following determination of risk level, high-risk youth will be assigned to complete ENCOMPASS, the comprehensive substance use treatment. Youth who are categorized as low- or mid-risk will be randomly assigned to one of the two brief interventions. Following randomization, youth will engage in either Teen Intervene or Family Check-Up with a brief intervention specialist.

##### Measuring treatment response

Following completion of the first round of brief interventions (three sessions of Teen Intervene), we will measure youth’s response to treatment. Treatment response will be measured utilizing the Brief Situational Confidence Questionnaire (BSCQ), an eight item self-report measure of one’s level of confidence in resisting urges to use substances in eight situations: unpleasant emotions, physical discomfort, pleasant emotions, testing of control, urges and temptations, conflict with others, social pressures, and pleasant times with others [42]. Individuals rate their confidence in abstaining from substance use in each of the situations on a 0% (*not at all confident*) to 100% (*completely confident*), with higher scores denoting greater confidence in abstaining from substance use. Treatment response will be determined utilizing a cut-off overall mean score of 70% on the BSCQ (i.e., average of 70% confidence in abstaining from using substance use across all eight scenarios), based on results from previous studies utilizing the BSCQ as a measure of symptom improvement [43]. The cutoff of 70% has two advantages: it is the average treatment response expected for this intervention, and it yields (approximately, if the distribution is symmetric) an anticipated equal sample-size split for responders and non-responders for the second randomization. Those with a post-intervention score of 70% or higher will be considered “treatment responders” and those whose BSCQ post-intervention score is lower than 70% will be considered “treatment non-responders.” Those youth who are considered “responders” will be told that they have successfully completed the intervention; they will complete additional post-treatment measures at 3 and 6 month follow-up. Non-responders will continue to the second randomization.

**Fig. 2:**
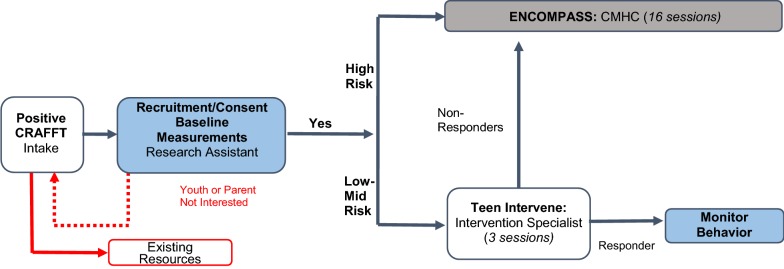
Overview of study design. YIJJ who score 1 or more on the CRAFFT will be recruited for the study. If interested and consent to treatment, they will be assessed to determine risk level. YIJJ identified as high-risk youth will be assigned to complete ENCOMPASS. Low to mid risk youth will be assigned to the brief intervention, Teen Intervene. Following the brief intervention, we will measure treatment response using the BSCQ. YIJJ who score below 70% will be considered “non-responders” and referred to ENCOMPASS

Following completion of treatment response measures using the BSCQ, those who are identified as “non-responders” (i.e., mean post-test BSCQ score lower than 70) will complete a second round of brief interventions. If youth continue to not respond, clinicians will make recommendations and referrals as appropriate. Following completion of the second round of brief intervention, treatment response will be measured again using the BSCQ and participants will complete 3- and 6- month follow-up (see Fig. 2).

#### Ongoing data collection

In addition to the intervention, participants and their caregivers will complete data collection at three time points: baseline, 3 months, and 6 months. Parents and youth each will be compensated $50 at each data collection point. In addition to measures of treatment response as described above, participants will complete self-report measures of family functioning, mental health symptoms, other risk behaviors, and psychosocial functioning (see Table 1 for overview of measures). These additional measures will help to explore additional characteristics related to treatment response.

Community partners will complete pre- and post-intervention measures related to feasibility of implementation and sustainability (e.g., organizational climate; see Table 1 for complete list of measures).

### Data analysis

#### Measurement of intervention effectiveness and treatment response

##### Preliminary analyses

Preliminary analyses will be used to compare the primary treatment response outcome (confidence of abstaining measured by the BSCQ) and substance use outcome across the responders and non-responders of the brief intervention and ENCOMPASS at baseline, 3 months, and 6 months. A separate model will be utilized to measure effectiveness among those assigned to ENCOMPASS. Substance use will be calculated as number of days in the past month of using alcohol, marijuana, and other substances (range 0–90). These outcomes will be compared between groups with longitudinal generalized linear models (GLM) using the GENMOD procedure with a linear link and normal error distribution. The BSCQ and substance use outcome will be analyzed in separate models. The baseline measure of BSCQ or substance use days will be adjusted as covariates and the repeated measurements of each outcome at 3 months and 6 months will be analyzed simultaneously in the model.

The GLMs are easily fit for non-Gaussian distributions by specifying appropriate link functions and error distributions. If substance use days exhibit sparse counts or large frequency of zeros, we will consider using logistic, Poisson, or zero-inflated Poisson regression models, depending on the observed distributions of outcome behaviors. We also will explore analyzing substance use as time-to-event data using a Cox proportional hazards regression model with the SAS PHREG procedure with censoring if loss to follow-up occurs. Effect sizes will be computed from the model as differences between groups on adjusted means divided by SDs for the linear models, or differences between odds for logistic models or hazards for Poisson models.

The median and relevant percentile (e.g. 25th or 75th) of time to events will be presented by group with associated confidence intervals. All models will include an indicator variable for whether the subject was enrolled as a detained or probation youth. Other covariates will be included based on their theoretical importance as potential confounders and on their imbalance between randomized arms at baseline. We also will examine moderators that we have hypothesized to be potential determinants of treatment response (e.g., family functioning).

##### Analyses

The specific analyses and hypothesis tests (which are preliminary because this is a feasibility study) will assess effectiveness of the brief intervention and ENCOMPASS on substance use outcomes. These data will be used to generate hypotheses about brief and comprehensive interventions for a future larger study.

##### Sample size and power

We plan to recruit n = 80 youth and caregiver dyads per county (160 total). We estimate that no more than 10% of youth in each county will be high-risk, resulting in a sample size of n = 16 assigned to ENCOMPASS and n = 144 to first-stage brief intervention (BI). We anticipate 20% loss at 6-month follow-up resulting in n = 12 in the ENCOMPASS group and n = 116 in the first-stage BI with 6 month data (58 responders and 58 non-responders, assuming a 50% response rate. We have accounted for a sample size inflation factor related to potential within-county intraclass correlation (ICC) of outcomes. Specifically, in the first step of the back-calculation, we determined that a sample size of n = 40 per responders and non-responders to the first-stage intervention (with 6-month data) is required under the “independence of observations” assumption (i.e., *before* accounting for clustering) to provide 80% power for the two-sided model-based tests to detect an effect size (0.63) that is between medium (0.50) and large (≥ 0.80). For a first-stage effective sample size of 40 in each group (responder vs. non-responder), there is a 1.39 sample size inflation factor (IF) when the ICC = 0.01 and the number of clusters (i.e., counties) = 2: IF = [1 + (average 40 patients per cluster − 1) × ICC of 0.01]. Therefore, an actual sample size of 56 per first-stage brief intervention (i.e., 40 × 1.39) with 6-month data is required (i.e., an effective sample size of 40 per first-stage brief intervention with 6-month data if observations were not clustered). The sample of 56 in each group will provide adequate power for this feasibility study and its preliminary hypothesis tests, given that we anticipate n = 58 per first-stage group (responder vs. non-responder) with 6-month data. This power calculation is conservative because the longitudinal models will have slightly increased power due to incorporating all available data (baseline, 3-months, 6-months), including data from persons missing one but not all times points. The goal will be to estimate effect sizes, for which the planned sample size will be adequate. A minimum of 12 per group is recommended for estimating effects sizes of continuous outcomes in pilot or feasibility studies [44].

#### Measurement of implementation in juvenile justice and CMHC settings

We will utilize both qualitative and quantitative methods to assess implementation of substance use screening in the juvenile justice systems and implementation of interventions in the CMHCs.

##### Stages of implementation completion exploratory analyses

We will utilize the stages of implementation completion (SIC) tool to track and assess implementation progress separately for the CMHCs and juvenile justice settings. Utilizing the online SIC tracker, we will log data occurring at each of the implementation stages: pre-implementation (exploration and preparation), implementation, and sustainability. The online SIC tracker computes SIC scores at each implementation stage for measures such as proportion (percentage of planned activities performed) and duration (number of days between activities), which have been shown to predict successful achievement of implementation milestones [45, 46]; data also provides insights into organizational implementation behavior predicting sustainment.

##### Quantitative analysis

We will conduct separate analyses for juvenile justice systems and CMHCs based on CMHC and juvenile justice staff completion of implementation measures (see Table 1 for complete list of measures). We will use linear mixed models to analyze repeatedly measured implementation variables (baseline/pre-implementation, 6 months/post-implementation) to determine whether implementation variables change over time. In one model measuring implementation of substance use screening in juvenile justice settings, organizational readiness to implement change (based on juvenile justice staff self-reports) will serve as the dependent variable and staff and setting characteristics (e.g., demographics, years of experience, see Table 1) will serve as independent variables. Similar analyses will be conducted to assess implementation with the CMHCs.

##### Qualitative analysis

We will conduct separate qualitative analyses for implementation of substance use screening in the juvenile justice systems and implementation of interventions in the CMHCs. We will separately analyze qualitative interviews with juvenile justice staff and CMHC staff using an inductive, interpretive approach based on grounded theory [47–49]. Two coders will initially review transcripts and complete open coding to identify emergent themes. Following open coding, coders will discuss findings and then complete focused coding utilizing a determined coding scheme based on emergent themes. This qualitative analysis can be viewed as a developmental approach where findings will both improve understanding of implementation and inform refinement of implementation strategies.

## Discussion

Youth in the juvenile justice system (YIJJ) report high rates of substance use and behavioral health problems; however, are less likely to receive services compared to their non-offending peers. For one, YIJJ often are not appropriately screened for substance use or other behavioral health problems; if they are identified as needing services, there are often not appropriate services available for youth in community-based settings. The project aims to address these barriers by utilizing a hybrid type 1 clinical effectiveness-implementation design to (1) implement universal validated screening for substance use in two county juvenile justice systems, and (2) implement a brief intervention and a comprehensive EBI to equip CMHCs with appropriate EBIs to target YIJJ youth with a range of substance use severity. In addition to testing the feasibility of implementing a brief intervention and a communication system between juvenile justice and CMHCs, we also will compare the effectiveness of a comprehensive intervention.

This study advances research on implementation studies of interventions for YIJJ by using a risk stratification approach which emphasizes that treatment should be individualized and least restrictive as multiple barriers and challenges exist to engage youth and families in treatment. Moreover, this approach should be helpful in geographic areas with workforce access issues. To date, few studies have examined the effectiveness of evidence-based brief interventions for substance use among YIJJ. Understanding their effectiveness could allow for more universal dissemination to other justice systems.

## Limitations

The project is not without limitations. For one, there is evidence that those youth with more severe substance use or behavioral health problems are even less likely to receive services due to a number of barriers, such as family functioning and environmental resources. Thus, given the voluntary nature of the study, it is possible that we will not be able to engage those particularly high-risk youth. Nonetheless, we will be utilizing a home-based model of care which should increase treatment engagement and eliminate barriers such as transportation. Another limitation is that turnover of case managers in community mental health centers is high; this significant turnover may limit sustainability of the interventions. The staffing cost of continued supervision and training to high fidelity is a barrier to sustainment of evidence-based treatment [50]. However, complex interventions, such as ENCOMPASS, are necessary with vulnerable populations presenting with multiple risk behaviors and diagnoses. Thus, sustainment and expansion of complex interventions are important goals for communities and gathering pilot data on the barriers to these interventions is needed to further this important work. Lastly, we are implementing these services in two smaller counties each with only one CMHC; this model of increasing communication and collaboration between the JJ system and the CMHC may be more challenging for larger counties with multiple organizations that offer treatment services.

## Supplementary information

**Additional file 1: Appendix S1.** The CRAFFT screening interview.

## Data Availability

Not applicable. Data collection has not started.

## Abbreviations

BSCQ: Brief Situational Confidence Questionnaire
CMHC: community mental health center
EBI: evidence-based intervention
EPIS: exploration, preparation, implementation, sustainability
SIC: stages of implementation completion
SUD: substance use disorders
YIJJ: youth involved in the juvenile justice system

## References

[1] Kessler RC, Avenevoli S, Costello EJ, Georgiades K, Green JG, Gruber MJ (2012). Prevalence, persistence, and sociodemographic correlates of DSM-IV disorders in the National Comorbidity Survey Replication Adolescent Supplement. Arch Gen Psychiatry.

[2] Shufelt J, Cocozza J. Youth with mental health disorders in the juvenile justice system: results from a multi-state prevalence study. National Center for Mental Health and Juvenile Justice; 2006. http://www.ncmhjj.com/pdfs/publications/PrevalenceRPB.pdf.

[3] Teplin LA, Abram KM, McClelland GM, Dulcan MK, Mericle AA (2002). Psychiatric disorders in youth in juvenile detention. Arch Gen Psychiatry.

[4] Vincent GM, Grisso T, Terry A, Banks S (2008). Sex and race differences in mental health symptoms in juvenile justice: the MAYSI-2 national meta-analysis. J Am Acad Child Adolesc Psychiatry.

[5] Wasserman GA, McReynolds LS, Schwalbe CS, Keating JM, Jones SA (2010). Psychiatric disorder, comorbidity, and suicidal behavior in juvenile justice youth. Crim Just Behav.

[6] Aalsma MC (2012). Use of outpatient care by juvenile detainees upon community reentry: effects of mental health screening and referral. Psychiatr Serv.

[7] Fazel S, Doll H, Långström N (2008). Mental disorders among adolescents in juvenile detention and correctional facilities: a systematic review and metaregression analysis of 25 surveys. J Am Acad Child Adolesc Psychiatry.

[8] Abram KM, Zwecker NA, Welty LJ, Hershfield JA, Dulcan MK, Teplin LA (2015). Comorbidity and continuity of psychiatric disorders in youth after detention: a prospective longitudinal study. JAMA Psychiatry..

[9] Hoeve M, McReynolds LS, Wasserman GA (2014). Service referral for juvenile justice youths: associations with psychiatric disorder and recidivism. Admin Policy Ment Health Ment Health Serv Res.

[10] Sickmund M, Puzzanchera C (2014). Juvenile offenders and victims: 2014 national report.

[11] Cuellar A, McReynolds LS, Wasserman GA (2006). A cure for crime: can mental health treatment diversion reduce crime among youth?. J Policy Anal Manag.

[12] Dopp AR, Borduin CM, White II, Mark H, Kuppens S (2017). Family-based treatments for serious juvenile offenders: a multilevel meta-analysis. J Consult Clin Psychol.

[13] Foster EM, Qaseem A, Connor T (2004). Can better mental health services reduce the risk of juvenile justice system involvement?. Am J Public Health.

[14] Henggeler SW, Schoenwald SK (2011). Evidence-based interventions for juvenile offenders and juvenile justice policies that support them. Social policy report.

[15] Pullmann MD, VanHooser S, Hoffman C, Heflinger CA (2010). Barriers to and supports of family participation in a rural system of care for children with serious emotional problems. Community Ment Health J.

[16] Sexton T, Turner CW (2010). The effectiveness of functional family therapy for youth with behavioral problems in a community practice setting. J Fam Psychol.

[17] Forgatch MS, Patterson GR. Parent management training—Oregon model: an intervention for antisocial behavior in children and adolescents. 2010.

[18] Young DW, Dembo R, Henderson CE (2007). A national survey of substance abuse treatment for juvenile offenders. J Subst Abuse Treat.

[19] Tubman JG, Gil AG, Wagner EF (2004). Co-occurring substance use and delinquent behavior during early adolescence: emerging relations and implications for intervention strategies. Criminal Justice Behav.

[20] Aalsma MC, Brown JR, Holloway ED, Ott MA (2014). Connection to mental health care upon community reentry for detained youth: a qualitative study. BMC Public Health..

[21] Sweeny K (2008). Crisis decision theory: decisions in the face of negative events. Psychol Bull.

[22] Dauria EF, McWilliams MA, Tolou-Shams M (2018). Substance use prevention and treatment interventions for court-involved, non-incarcerated youth. Brief Intervent Adolesc Alcohol Substance Abuse..

[23] Morse G, Salyers M, Rollins A, Monroe-DeVita M, Pfahler C (2012). Burnout in mental health services: a review of the problem and its remediation. Admin Policy Ment Health Ment Health Serv Res.

[24] Paris M, Hoge MA (2010). Burnout in the mental health workforce: a review. J Behav Health Serv Res.

[25] Mee-Lee D, Shulman GD, Fishman MJ, Gastfriend DR, Miller MM (2013). The ASAM criteria: treatment criteria for addictive, substance-related, and co-occurring conditions.

[26] Barnett ML, Lau AS, Miranda J (2018). Lay health worker involvement in evidence-based treatment delivery: a conceptual model to address disparities in care. Ann Rev Clin Psychol.

[27] Winters KC, Fahnhorst T, Botzet A, Lee S, Lalone B (2012). Brief intervention for drug-abusing adolescents in a school setting: outcomes and mediating factors. J Subst Abuse Treat.

[28] Winters KC, Lee S, Botzet A, Fahnhorst T, Nicholson A (2014). One-year outcomes and mediators of a brief intervention for drug abusing adolescents. Psychol Addict Behav.

[29] Dembo R, Briones Robinson R, Schmeidler J, Wareham J, Ungaro R, Winters KC, Belenko S (2014). Brief intervention impact on truant youths’ marijuana use: 18-month follow-up. J Child Adolesc Subst Abuse.

[30] Hogue A, Henderson CE, Becker SJ, Knight DK (2018). Evidence base on outpatient behavioral treatments for adolescent substance use, 2014–2017: outcomes, treatment delivery, and promising horizons. J Clin Child Adolesc Psychol.

[31] Thurstone C, Riggs PD, Salomonsen-Sautel S, Mikulich-Gilbertson SK (2010). Randomized, controlled trial of atomoxetine for attention-deficit/hyperactivity disorder in adolescents with substance use disorder. J Am Acad Child Adolesc Psychiatry..

[32] Riggs PD, Mikulich-Gilbertson SK, Davies RD, Lohman M, Klein C, Stover SK (2007). A randomized controlled trial of fluoxetine and cognitive behavioral therapy in adolescents with major depression, behavior problems, and substance use disorders. Arch Pediatr Adolesc Med.

[33] Riggs PD, Winhusen T, Davies RD, Leimberger JD, Mikulich-Gilbertson S, Klein C, Macdonald M, Lohman M, Bailey GL, Haynes L, Jaffee WB (2011). Randomized controlled trial of osmotic-release methylphenidate with cognitive-behavioral therapy in adolescents with attention-deficit/hyperactivity disorder and substance use disorders. J Am Acad Child Adolesc Psychiatry.

[34] Feder KA, Krawczyk N, Saloner B (2017). Medication-assisted treatment for adolescents in specialty treatment for opioid use disorder. J Adolesc Health.

[35] Committee OS (2016). Medication-assisted treatment of adolescents with opioid use disorders. Pediatrics.

[36] Fishman MJ, Winstanley EL, Curran E, Garrett S, Subramaniam G (2010). Treatment of opioid dependence in adolescents and young adults with extended release naltrexone: preliminary case-series and feasibility. Addiction.

[37] Curran GM, Bauer M, Mittman B, Pyne JM, Stetler C (2012). Effectiveness-implementation hybrid designs: combining elements of clinical effectiveness and implementation research to enhance public health impact. Med Care.

[38] Aarons GA, Hulbert M, Horwitz SM (2011). Advancing a conceptual model of evidence-based practice implementation in public service sectors. Admin Policy Ment Health Serv Res.

[39] Chamberlain P, Brown CH, Saldana L (2011). Observational measure of implementation progress in community based settings: the stages of implementation completion (SIC). Implement Sci.

[40] Saldana L (2014). The stages of implementation completion for evidence-based practice: protocol for a mixed methods study. Implement Sci.

[41] Saldana L, Chamberlain P, Wang W, Brown CH (2012). Predicting program start-up using the stages of implementation measure. Admin Policy Ment Health Ment Health Serv Res.

[42] Knight JR, Sherritt L, Harris SK, Gates EC, Chang G (2003). Validity of brief alcohol screening tests among adolescents: a comparison of the AUDIT, POSIT, CAGE, and CRAFFT. Alcohol Clin Exp Res..

[43] Sobell LC, Cunningham JA, Sobell MB, Agrawal S, Gavin DR, Leo GI, Singh KN (1996). Fostering self-change among problem drinkers: a proactive community intervention. Addict Behav.

[44] Slavet JD, Stein LA, Klein JL, Colby SM, Barnett NP, Monti PM (2005). Piloting the family check-up with incarcerated adolescents and their parents. Psychol Serv..

[45] Julious SA (2005). Sample size of 12 per group rule of thumb for a pilot study. Pharm Stat.

[46] Charmaz K (2006). Constructing grounded theory: a practical guide through qualitative analysis.

[47] Lang JM, Connell CM (2017). Measuring costs to community-based agencies for implementation of an evidence-based practice. J Behav Health Serv Res..

[48] Ehrhart MG, Aarons GA, Farahnak LR (2015). Going above and beyond for implementation: the development and validity testing of the Implementation Citizenship Behavior Scale (ICBS). Implement Sci.

[49] Ehrhart MG, Aarons GA, Farahnak LR (2014). Assessing the organizational context for EBP implementation: the development and validity testing of the Implementation Climate Scale (ICS). Implement Sci.

[50] Aarons GA, Ehrhart MG, Farahnak LR (2014). The implementation leadership scale (ILS): development of a brief measure of unit level implementation leadership. Implement Sci.

[51] Shea CM, Jacobs SR, Esserman DA, Bruce K, Weiner BJ (2014). Organizational readiness for implementing change: a psychometric assessment of a new measure. Implement Sci.

[52] Athay MM, Bickman L (2012). Development and psychometric evaluation of the youth and caregiver service satisfaction scale. Admin Policy Ment Health Ment Health Serv Res.

[53] Kelly SM, Gryczynski J, Mitchell SG, Kirk A, O’Grady KE, Schwartz RP (2014). Validity of brief screening instrument for adolescent tobacco, alcohol, and drug use. Pediatrics..

[54] Currie C, Molcho M, Boyce W, Holstein B, Torsheim T, Richter M (2008). Researching health inequalities in adolescents: the development of the Health Behaviour in School-Aged Children (HBSC) family affluence scale. Soc Sci Med.

[55] Meneses-Gaya CD, Zuardi AW, Loureiro SR, Crippa JA (2009). Alcohol Use Disorders Identification Test (AUDIT): an updated systematic review of psychometric properties. Psychol Neurosci.

[56] Huebner AJ, Howell LW (2003). Examining the relationship between adolescent sexual risk-taking and perceptions of monitoring, communication, and parenting styles. J Adolesc Health.

[57] Victor SE, Klonsky ED (2016). Validation of a brief version of the difficulties in emotion regulation scale (DERS-18) in five samples. J Psychopathol Behav Assess..

[58] Scott BG, Lapré GE, Marsee MA, Weems CF (2014). Aggressive behavior and its associations with posttraumatic stress and academic achievement following a natural disaster. J Clin Child Adolesc Psychol.

[59] Lippman LH, Moore KA, McIntosh H (2011). Positive indicators of child well-being: a conceptual framework, measures, and methodological issues. Appl Res Qual Life..

