# Supplementary material for: Implementing risk stratification to the treatment of adolescent substance use among youth involved in the juvenile justice system: protocol of a hybrid type I trial
Source: Addict Sci Clin Pract. 2019 Sep 6;14:36. doi: 10.1186/s13722-019-0161-5 (PMC6729049; doi:10.1186/s13722-019-0161-5)
Supplement: Supplementary file 1 — Additional file 1: Appendix S1. The CRAFFT screening interview. [file 13722_2019_161_MOESM1_ESM.docx]

**Appendix A.**

| **The CRAFFT Screening Interview** | | |
| --- | --- | --- |
| Begin: *“I’m going to ask you a few questions that I ask all my patients. Please be honest. I will keep your answers confidential.”* | | |
| ***Part A*** |  |  |
| **During the PAST 12 MONTHS, did you:** | **No** | **Yes** |
| **1**. Drink any alcohol (more than a few sips)? | 󠄚 | 󠄚 |
| **2**. Smoke any marijuana or hashish? | 󠄚 | 󠄚 |
| **3**. Use anything else to get high? | 󠄚 | 󠄚 |
| (“anything else” includes illegal drugs, over the counter and prescription drugs, and things that you sniff or “huff”) | | |
| *Did the individual answer “yes” to any questions in part A?* | | |
| If NO, ask CAR question only, then stop | If YES, ask all 6 questions | |
| ***Part B*** | **No** | **Yes** |
| **1.** Have you ever ridden in a **CAR** driven by someone (including yourself) who was “high” or had been using alcohol or drugs? | 󠄚 | 󠄚 |
| **2.** Do you ever use alcohol or drugs to **RELAX**, feel better about yourself, or fit in? | 󠄚 | 󠄚 |
| **3.** Do you ever use alcohol or drugs while you are by yourself, or **ALONE**? | 󠄚 | 󠄚 |
| **4.** Do you ever **FORGET** things you did while using alcohol or drugs? | 󠄚 | 󠄚 |
| **5.** Do your **FAMILY** or **FRIENDS** ever tell you that you should cut down on your drinking or drug use? | 󠄚 | 󠄚 |
| **6.** Have you ever gotten into **TROUBLE** while you were using alcohol or drugs? | 󠄚 | 󠄚 |
